# The association between bed occupancy rates and hospital quality in the English National Health Service

**DOI:** 10.1007/s10198-022-01464-8

**Published:** 2022-05-17

**Authors:** Laia Bosque-Mercader, Luigi Siciliani

**Affiliations:** Department of Economics and Related Studies, University of York, York, YO10 5DD UK

**Keywords:** Bed occupancy rates, Hospital quality, National Health Service, England, I10, I11

## Abstract

We study whether hospitals that exhibit systematically higher bed occupancy rates are associated with lower quality in England over 2010/11–2017/18. We develop an economic conceptual framework to guide our empirical analysis and run regressions to inform possible policy interventions. First, we run a pooled OLS regression to test if high bed occupancy is associated with, and therefore acts as a signal of, lower quality, which could trigger additional regulation. Second, we test whether this association is explained by exogenous demand–supply factors such as potential demand, and unavoidable costs. Third, we include determinants of bed occupancy (beds, length of stay, and volume) that might be associated with quality directly, rather than indirectly through bed occupancy. Last, we use a within-between random-effects specification to decompose these associations into those due to variations in characteristics between hospitals and variations within hospitals. We find that bed occupancy rates are positively associated with overall and surgical mortality, negatively associated with patient-reported health gains, but not associated with other indicators. These results are robust to controlling for demand–supply shifters, beds, and volume. The associations reduce by 12%-25% after controlling for length of stay in most cases and are explained by variations in bed occupancy between hospitals.

## Introduction

Policymakers aim at improving quality of care and efficiency of health systems. Aligning both objectives may be difficult and a trade-off might arise [1]. Within the hospital sector, one major concern relates to the increasingly intense use of beds that leads to higher bed occupancy rates (the ratio of the number of occupied beds over available beds), and therefore efficiency, but potentially lower quality [2].

Bed occupancy rates have increased due to secular declines in beds and a growing demand for hospital services. Hospital beds per capita reduced in most OECD countries from an average of 5.8 per 1000 population in 2000 to 4.7 in 2017 [3]. Several factors drove this reduction. First, progress in medical technology allowed countries to perform more surgeries on a same-day basis avoiding overnight stays [3] and shortening length of stay (LOS). LOS also reduced under the pressure to cut costs induced by prospective payment systems based on Diagnosis-Related Group (DRG) tariffs [3] and programmes such as the English Reducing Length of Stay [4]. Second, reduction in hospital capacity was accelerated by cuts in public health spending following the financial and economic crises in European countries^1^ [5] and broader policies aimed at reducing hospital admissions [3]. These supply changes were accompanied by a growing demand for beds linked to the rising prevalence of chronic conditions and an ageing population [2].

Low bed occupancy may be a sign of underutilisation and leave scope for improving efficiency. However, high bed occupancy rates may also be problematic if they are symptomatic of a health system under pressure and result in inappropriate and undesirable practices that lead to premature discharges, overcrowding of facilities, staff workload pressure, and eventually worse quality of care (see “Conceptual framework” for a detailed discussion).

Due to COVID-19, countries had to suspend planned care and a backlog of patients was formed as a result. Given the limited capacity that several health systems face, the high demand for healthcare from the backlog is likely to put pressure on hospitals to increase bed occupancy rates. It is, therefore, important to understand the relation between bed occupancy rates and hospital quality.

According to the National Audit Office [6], bed occupancy rates are deemed efficient if around 85%, while rates above this level might lead to periodic bed shortages and levels exceeding 90% may prompt regular bed crises [7]. Although costly, maintaining some beds unoccupied is necessary to ensure hospitals can meet unexpected demand and deliver good quality of care [7].

**Fig. 1:**
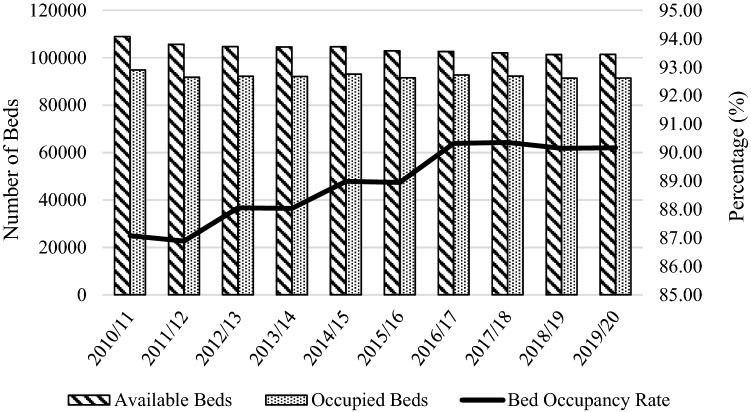
General and acute available and occupied beds and bed occupancy rates (2010/11–2019/20). Source: NHS England Statistics (https://www.england.nhs.uk/statistics/statistical-work-areas/bed-availability-and-occupancy/)

This is the case of the National Health Service (NHS) in England where concerns related to declines in the number of beds and increases in bed occupancy rates have been raised [8]. The number of overnight general and acute beds fell by 7% between 2010/11 and 2019/20, while occupied beds only decreased by 4%.^2^ As a result, general and acute bed occupancy increased from 87 to 90% over the same period (Fig. 1).

Despite its policy relevance, evidence on the association between high bed occupancy rates and hospital quality is limited and inconclusive (see “Related literature”). The aim of this study is to investigate if hospitals that exhibit systematically higher bed occupancy rates in the English NHS are *associated* with lower quality and whether a range of demand–supply factors and determinants of bed occupancy rates can explain such association. Although our estimates cannot be interpreted as causal, they inform possible policy interventions as explained below.

We first develop a conceptual framework of the intricate relation between bed occupancy rates and hospital quality. We show how a range of demand–supply factors affect both bed occupancy and quality. We give special attention to three variables of which bed occupancy rates are a function (beds, LOS, and volume of patients treated) and explain how these affect quality both directly and indirectly through bed occupancy, while being themselves affected by demand–supply factors.

Our conceptual framework guides the empirical analysis. First, we run a pooled regression of quality on bed occupancy rates only controlling for year fixed effects. This allows us to test if bed occupancy is associated and therefore acts as a signal of lower quality. If this is the case, then regulators could use high bed occupancy rates as an indicator to trigger additional monitoring or auditing interventions on hospital quality. In this respect, it is important not to control for other factors in the empirical analysis, as the regulator would want to address low quality regardless of the factors causing it.

Second, we test if any association between bed occupancy rates and quality is explained by exogenous demand factors (e.g. elderly population, income deprivation) and supply factors (e.g. unavoidable costs, skill mix, type of hospital). This might help regulators to cluster groups of hospitals based on the characteristics of the population in the catchment area they serve (e.g. deprived areas) or hospital characteristics (e.g. high unavoidable labour and capital costs or teaching status).

Third, we further include three key determinants of bed occupancy rates that might be associated with quality directly and indirectly, which in our conceptual framework have shown to be LOS, volume, and beds. This specification allows identifying which source of variation in bed occupancy rates is responsible for the association with quality. For example, high bed occupancy rates may be driven by high LOS, high volume of admissions, low availability of beds or a combination of them.

Fourth, we estimate a within–between random-effects model to decompose the association between quality and bed occupancy that is due to the time-invariant component of bed occupancy rates across hospitals (*between* association) versus the time-varying component of bed occupancy rates (*within* association). This approach allows to inform possible policy interventions. For example, if we find that the association is due to variation *between* hospitals, then regulators can target hospitals that systematically perform poorly. If instead variation arises *within* hospitals, then regulators can target hospitals experiencing sharp increases in bed occupancy rates over time, even when starting at lower levels of bed occupancy rates. The advantage of the within–between random-effects model is that it allows to explore simultaneously both variations in bed occupancy rates over time (within association), and variations across providers (between association). This latter would be precluded in a fixed effect model because the variations in characteristics across providers (between variation) would be absorbed by the hospital fixed effects.

Our data comprise a wide range of risk-adjusted quality measures (overall mortality, surgical and condition-specific—heart attack, hip fracture, and stroke—mortality, emergency readmission rates, and patient-reported health outcomes for hip and knee replacements) and overnight bed occupancy rates for English public acute hospitals over 2010/11–2017/18.

The results show that bed occupancy rates are negatively associated with a subset of quality indicators. In more detail, bed occupancy rates are positively associated with overall and surgical mortality (higher mortality implies lower quality) and negatively associated with patient-reported health outcomes for hip and knee replacements, while they are not associated with condition-specific mortality nor emergency readmissions. In quantitative terms, a 5 percentage points (p.p.) increase in bed occupancy is associated with 0.5%–0.9% reduction in patient-reported health outcomes, 1.1% increase in overall mortality, and 3.1% increase in surgical mortality. We focus on a 5p.p. increase in bed occupancy rate as this corresponds to about one standard deviation observed in the data. These associations are not explained by demand–supply shifters, nor by hospital availability of beds or patient volume. Instead, LOS explains 12%–25% of the association between bed occupancy and overall and surgical mortality, and health gain after a knee replacement. Finally, these associations are explained by variations in bed occupancy rates between hospitals rather than within hospitals, except for surgical mortality, therefore suggesting that these associations are persistent over time across hospitals.

The study makes different contributions to the literature. First, we provide a novel conceptual framework, which highlights the complex relation between bed occupancy rates, quality and its supply and demand determinants. Second, this conceptual framework guides our empirical analysis, which is used to answer four policy-related questions that can help regulators tackling low quality associated with high bed occupancy rates. Unlike previous evidence, we do not only aim at estimating the association between bed occupancy rates and hospital quality, but we explore factors that might explain it. Third, we extend previous work [1, 9–15] with a richer set of quality measures, such as condition-specific mortality and Patients Reported Outcome Measures for knee and hip replacements, and a wider set of control variables, such as hospital competition, unavoidable costs (Market Forces Factor), and characteristics of population residing in the hospital’s catchment area. We also focus on a long panel of data for a time period (2010–18) characterised by high bed occupancy rates between 85% and 90%. Fourth, we decompose the association between bed occupancy and quality that is due to variations in bed occupancy both *across* and *within* hospitals using a within–between random-effects model. Last, we emphasise the role of LOS in explaining the association between quality and bed occupancy rates.

The rest of this study is structured as follows. “Related literature” reviews the literature and “English National Health Service” gives the institutional background. “Conceptual framework” develops the conceptual framework. “Econometric approach” outlines the regression methods. “Data” describes the data and “Results” provides and discusses the results. “Conclusion” concludes.

### Related literature

Our study contributes to the literature on the association between bed occupancy and quality and, more broadly, to the literature on the relation between efficiency and quality. Several clinical studies investigate the association between bed occupancy rates and hospital quality with mixed findings. Some find a positive association between bed occupancy and in-hospital mortality and mortality following discharge from hospital in Western Australia [15], Germany [1], and Denmark [14]. On the contrary, Long and Mathews [13] find a negative association between ward occupancy rates and in-hospital mortality for the United States. Boden et al. [11] analyse an intervention that aimed at reducing bed occupancy to 90% over a 32-month period at an English hospital trust applying interrupted time-series analysis. They show that lowering medical bed occupancy is associated with a decrease in mortality.

For Sweden, Blom et al. [9, 10] evaluate the association between bed occupancy rates and unplanned 72 h revisits to the emergency department and emergency readmissions within 30 days of hospital discharge, respectively. The former study finds no significant association, while the latter finds a positive association. Friebel et al. [12] use a two-year panel of data comprising all non-specialist acute hospital trusts in England and find a small clinically significant positive association between bed occupancy rates and emergency readmissions after controlling for hospital fixed effects.^3^^,^^4^

### English National Health Service

The English NHS provides healthcare free at the point of use. It is publicly funded through general taxation and monitored by the Department of Health and Social Care. Health expenditure per capita in nominal values increased by 115% from £891 in 2000/01 to £1,912 in 2012/13, although annual growth decreased from 10% between 2000/01 and 2010/11 to 1% between 2010/11 and 2012/13 [16]. Annual growth in health expenditure per capita was on average 3% until 2018/19 [17, 18].

General practitioners provide primary care and act as gatekeepers to access specialist services. NHS patients can attend both public and private hospitals. Public hospitals are aggregated in organisational units called NHS Trusts,^5^ which can have teaching status by offering teaching and research activities and/or specialist status by focusing on particular conditions [19]. NHS Trusts might also have Foundation Trust status obtaining more financial autonomy [20].

The English NHS has a prospective payment system known as Payment by Results since 2003/04 [20, 21] based on the Healthcare Resource Groups (HRGs), similar to DRGs in the United States. Patients can choose hospital which has fostered competition since 2008 [22].

Overnight acute beds fell from 110,568 to 102,194 between 2010/11 and 2019/20 [23]. Contributors to this decline are technology advances in medical care, such as day surgeries and improvements in anaesthetic and surgical procedures, pain control, and recovery methods, which led to reductions in LOS from 8.2 days in 2000/01 to 4.5 in 2018/19, and policies targeting at moving mental health, learning disabilities, and long-term care away from hospitals to community, and care, nursing, and patient’s homes [8]. Despite these efforts, hospital demand and admissions have continuously risen [24].

NHS England and NHS Improvement recommended to avoid bed occupancy rates above 92% [25]. The 2020/21 NHS national planning guidance stated a maximum of 92% to be achieved through increasing acute bed stocks, community care, investment in primary care, and reductions in LOS and admissions [26].

## Conceptual framework

**Fig. 2:**
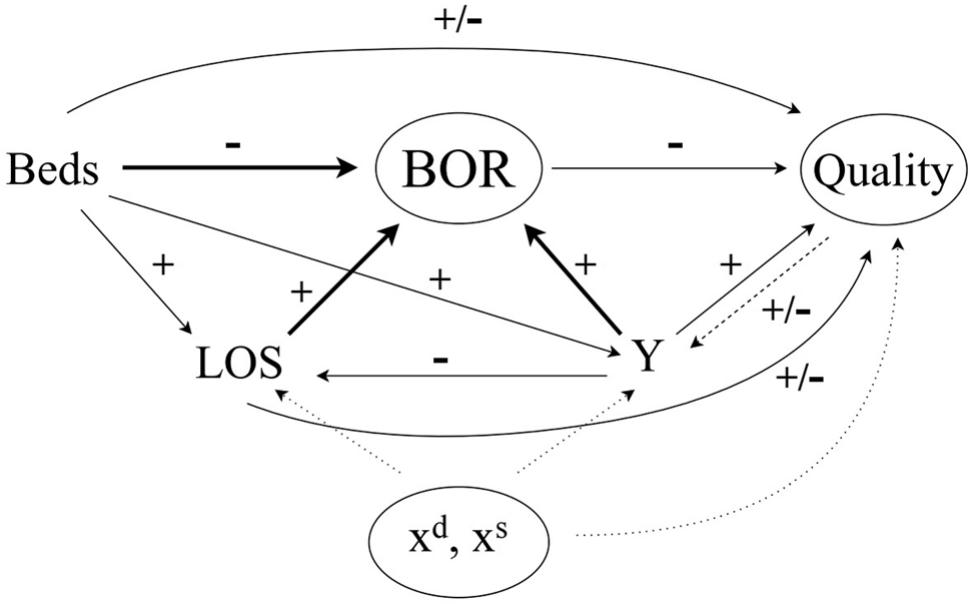
Conceptual framework. *BOR*  bed occupancy rate; *LOS*  length of stay, *Y*  volume, *x*^*d*^  demand shifters, *x*^*s*^  supply shifters

We provide a conceptual framework on the relation between bed occupancy rates and quality. We distinguish between factors through which bed occupancy affects quality directly, and factors that affect both bed occupancy rates and quality. These relations are summarised in Fig. 2.

We define bed occupancy rate (*BOR*) in a given hospital in a given day as the number of *occupied* beds over the number of *available* beds:

$$BOR=\frac{occupied \, beds}{available \, beds} .$$

Assume for simplicity that patients do not differ in severity and have the same LOS, and that the system is in steady state so that the number of beds, occupied or available, and LOS are constant over time. For a given LOS (also measured in days) and number of occupied beds in a given day, the number of patients finishing treatment and being discharged *Y* each day in a given hospital is equal to $Y=\frac{occupied \, beds}{LOS}$. For example, if 90 beds are occupied each day (giving 90 occupied bed-days) and each patient stays three days, then on average 30 patients complete the treatment and are discharged in the hospital each day. We can, therefore, rewrite *BOR* as:

1 $${BOR}={ LOS}\times \frac{Y}{B}$$

where *B* is the number of available beds and *Y/B* is the ratio of volume of patients discharged over available beds. Bed occupancy rate is therefore determined by beds, LOS, and volume (bold arrows in Fig. 2).^6^

Hospital volume can be thought as the equilibrium between hospital demand and supply. Formally, volume *Y* = *Y(x*^*d*^*, x*^*s*^*, B, q)* is a function of demand shifters *x*^*d*^, supply shifters *x*^*s*^ (including hospital beds *B*), and quality *q*. Higher quality may affect equilibrium volume through both demand and supply (dashed arrow in Fig. 2). If patients can choose hospital, higher quality may attract more patients, but higher quality is also costly and implies lower supply and volume. On the demand side, providers respond to higher demand, due to an older or sicker population around the catchment area, by increasing supply and volume. Hospitals increase supply through bed expansions, re-organising staff shifts, hiring temporary staff, or speeding patients’ discharge. Supply shifters such as clinical staff and its composition, operating theatres and available beds^7^ increase volume.

We assume that LOS is a function of demand and supply shifters, including beds, and volume, *LOS = LOS*(*x*^*d*^*, x*^*s*^*, B, Y*). Higher availability of beds frees up capacity and might induce providers to increase LOS. Similarly, larger hospitals may treat more severe patients whose stays are longer. Higher demand or volume instead could induce providers to reduce LOS to accommodate additional patients, for a given capacity.

Our interest is in understanding the relation between quality and bed occupancy rates given by:

2 $$q=f\left({x}^{d}, {x}^{s},B,{ LOS}(.),Y(.),{ BOR}\left(B,{LOS}(.),Y(.)\right)\right) $$

where recall *LOS = LOS*(*x*^*d*^*, x*^*s*^*, B, Y*) and *Y* = *Y*(*x*^*d*^*, x*^*s*^*, B, q*)*.* Quality can be affected directly by bed occupancy rates, which are a function of beds, LOS, and volume, and by other factors, such as demand and supply shifters. Below, we describe these effects illustrated in Fig. 2.

### Direct effect of bed occupancy rates on quality

High bed occupancy rates imply limited availability of beds that can result in restricted access, which puts quality at risk [27]. First, assessment and treatment initiated in emergency wards and inappropriate admissions (e.g. allocating patients in unsuitable wards) might be more frequent. Second, admissions may be delayed, elective operations cancelled, and waiting times and trolley waits lengthened [28]. Third, hospitals might discharge patients prematurely [10, 12] to accommodate new admissions. This could shorten medical attention and incomplete treatments that may slow down patient’s health recovery, jeopardise patient’s care, and worsen health outcomes by increasing unplanned readmissions or deaths following hospital discharge.

Clinical staff face higher workloads when bed occupancy is high. This may imply more medical negligence and adverse events [29, 30], staff physical and mental fatigue [31], and greater ease of acquiring infections due to decreased hand-hygiene compliance, patient and staff movement, and less rigorous decontamination [32]. These malpractices might affect patients’ health and decline quality standards. Other reasons for hospital-acquired infections due to high bed occupancy rates include closer proximity between patients, reduced levels of patient cohorting (i.e. grouping patients exposed/diagnosed with a specific infection), and overburdening of isolation facilities [32]. Patients acquiring a hospital infection can see their condition aggravated.

### Demand and supply shifters

Demand and supply shifters (*x*^*d*^*, x*^*s*^) can affect quality directly, but also indirectly through the determinants of bed occupancy (i.e. LOS and volume, dotted arrows in Fig. 2).

Concerning demand shifters, hospitals located in more populated areas face larger demands which translate into higher volume but also shorter LOS, with an ambiguous effect on bed occupancy. Hospitals facing populations with higher need and degree of frailty (older, sicker or poorer) could translate into worse health outcomes and may affect bed occupancy rates through longer stays and higher volume.

Regarding supply shifters, hospitals with higher capital endowment (more MRI machines, CT scans) and labour endowment (more skilled workforce) may improve quality through better treatment and diagnosis and affect bed occupancy via volume and LOS. Providers with better management can enhance quality standards [33] and manage beds more efficiently. Hospitals facing more competition may attract patients by providing better quality [34] and experience higher bed occupancy. However, competition might also foster hospitals’ efficiency [35] by shortening stay, for given volume and beds. Providers may differ in exogenous (unavoidable) costs due to location that could reduce quality and put pressure on hospital’s LOS. Finally, teaching hospitals have a better reputation and their status is a marker of quality, while obtaining synergies through teaching and research.

### Beds, LOS and volume

Beds, LOS and volume affect bed occupancy rates by definition as shown in (1), but can directly influence quality (arrows from *Beds*, *LOS* and *Y* to *Quality* in Fig. 2).

A longer LOS might give patients more medical attention in a safer environment that could improve health status, but also wider exposure to infections and trigger mental health problems associated with hospitalisation. Hospitals with larger volume and capacity can exploit scale economies or learning-by-doing effects [36]. Larger hospitals can likewise benefit from scope economies by treating a broader range of diagnoses or technological advances that enable hospitals to be more productive by relying on new treatments and medical equipment.

## Econometric approach

To investigate the association between bed occupancy rates and quality, we estimate the following pooled OLS model:

3 $${q}_{ht}\, =\, {\beta }_{0}+{\beta }_{1}{{Occupancy}}_{ht}+{{\varvec{X}}}_{ht}^{{\prime}}{{\varvec{\beta}}}_{2}+{{\varvec{\lambda}}}_{t}+{\varepsilon }_{ht}$$

where *q*_*ht*_ is quality for hospital *h* in financial year *t* proxied by risk-adjusted health outcome measures (see “Data” for details), *Occupancy*_*ht*_ is bed occupancy rate, ***X***_*ht*_ is a vector of control variables related to demand (e.g. proportion of elderly, income deprivation), supply (e.g. labour endowment, unavoidable costs), hospital type (e.g. teaching status), and determinants of bed occupancy rates (beds, LOS, and volume), ***λ***_*t*_ is a vector of year fixed effects (e.g. to control for advances in technology), and *ε*_*ht*_ is the error term. The coefficient of interest is *β*_1_, which measures the association between quality and bed occupancy rate. We cluster standard errors at trust level to allow for serial correlation within hospitals.

Our specifications are guided by the conceptual framework in “Conceptual framework” and we estimate several versions of Eq. (3). First, we include no control variables except for year fixed effects, which we label Model 1. This shows whether bed occupancy is associated with lower quality and therefore acts as a signal of poor performance for a funder or regulator. Suppose that the association is strong. Whenever a regulator observes high bed occupancy rates, this regulator can infer that quality is more likely to be lower, and this is a reason to trigger some regulatory intervention in the form of additional monitoring or auditing. In this specification, we do not control for third factors since the regulator would want to tackle low hospital quality regardless of the factors causing it.

Second, we investigate the extent to which any association between bed occupancy rates and quality is explained by exogenous demand–supply factors. We follow the approach of Cutler & Lleras-Muney [37], who decomposed the health and education gradient. We add a set of explanatory variables that might be related to both bed occupancy and quality and compute the percentage decline in *β*_1_ from each variable that explains the association. We enter exogenous determinants of hospital demand (*x*^*d*^) and supply (*x*^*s*^) that could explain this association in Model 2. For example, hospitals serving an older population may face a higher demand with worse health status that reduces quality and have higher bed occupancy rates due to higher volume and longer stays. In addition, providers with higher (unavoidable) costs might have lower quality and respond by shortening LOS, which decreases bed occupancy rates. This analysis can help regulators to identify hospitals with both high bed occupancy rates and low quality that relate to the population living in the hospital’s catchment area or hospital features.

Third, we add sequentially factors that determine bed occupancy, as suggested by our conceptual framework, that might be associated with quality directly and indirectly through the correlation with bed occupancy rates. We include beds in Model 3 (which is also a supply shifter). Given that beds and volume (proxied by inpatients) are highly collinear (see “Data”), Model 4 includes beds and volume to beds ratio (*Y/B*—proxied by inpatients to beds ratio) where the latter can be thought as an indicator of technical efficiency. Instead, Model 5 adds only LOS to Model 3. These specifications allow identifying which determinant of bed occupancy rates is responsible for the association with quality.^8^

Finally, we decompose the association due to variations in bed occupancy *between* hospitals and variations over time *within* hospitals. We do so by estimating a within–between random-effects specification [38, 39] in Model 6. This model is closely linked to the “correlated random-effects” model by Mundlak [40] and Wooldridge [41]. This hybrid model replaces all time-variant independent variables (i.e. *Occupancy*_*ht*_, ***X***_*ht*_, ***λ***_*t*_) with their hospital-specific means over time (*Occupancy*_*h*_, ***X***_*h*_, ***λ***) and deviations from their mean (*Occupancy*_*ht*_–*Occupancy*_*h*_*, ****X***_*ht*_*–X*_*h*_, ***λ***_*t*_***–λ***). Standard errors are also clustered at trust level. Then, the within–between random-effects model is^9^:

4 $${q}_{ht}={\alpha }_{0}+{\alpha }_{1}Occupanc{y}_{h}+{\alpha }_{2}\left(Occupanc{y}_{ht}-Occupanc{y}_{h}\right)+{{\varvec{Z}}}_{h}^{{\prime}}{\boldsymbol{\alpha }}_{3}+({{\varvec{Z}}}_{ht}-{{{\varvec{Z}}}_{h}){^{\prime}}\boldsymbol{\alpha }}_{4}+{\varepsilon }_{ht} $$

where ***Z***_*h*_ includes ***X***_*h*_ and ***λ***, and ***Z***_*ht*_ includes ***X***_*ht*_ and ***λ***_*t*_.

*α*_1_ gives the association between quality and bed occupancy that is due to the time-invariant component of bed occupancy, i.e. the extent to which bed occupancy rates vary across hospitals (*between* association). This interpretation is in line with the pooled regression model in Eq. (3) and the related coefficient *β*_1_.

Instead, *α*_2_ gives the association between quality and bed occupancy that is due to the time-varying component of bed occupancy, i.e. the extent to which bed occupancy varies within hospitals over time (*within* association). This coefficient is the one that would be estimated with a fixed effects model, which controls for hospital fixed effects. The advantage of the within–between random-effects model is that it allows to explore within associations over time, while preserving the coefficients of the between associations. These would be precluded in a fixed effect model because any time-invariant hospital-specific mean variable (e.g. *Occupancy*_*h*_, ***X***_*h*_) would be absorbed by the hospital fixed effects.^10^

In economic terms, if the association is due to variation between hospitals, then regulators can target hospitals that systematically perform poorly. If instead the variation is within hospitals, then hospitals experiencing sharp increases in bed occupancy rates over time may be the source of concern.

In a robustness check, we test for possible non-linearities and estimate models where bed occupancy is measured as a vector of four categories: ≤ 85%, 85%–90%, 90%–95%, and > 95%, with 85%–90% used as the baseline category. Bed occupancy rate is deemed efficient at 85% [6] and some institutions recommend not to exceed 90% [42]. This specification tests whether hospitals with bed occupancy rates above 85% and 90% might experience longer delays in admissions and put patients’ health at a higher risk.

To summarise, although *β*_1_*,* *α*_1_ and *α*_2_ do not have a causal interpretation due to endogeneity problems such as simultaneous causality (volume might be affected by quality), the specifications outlined above can provide valuable insights to regulators in relation to using bed occupancy rates as a signal of quality and the factors behind such association.

## Data

The dataset is a panel of English NHS acute hospital trusts for 2010/11–2017/18. We exclude all non-acute (e.g. mental health providers) and specialist hospitals trusts (e.g. orthopaedics trusts) to homogenise our sample. The data are measured annually at the hospital trust level. Detailed variables’ definitions and sources are in Table 4 in the Appendix.

### Dependent variables

We include four type of quality indicators available from NHS Digital, which are measured at the hospital level and are already risk-adjusted for hospital case-mix^11^: Summary Hospital-level Mortality Indicator (SHMI), surgical and condition-specific mortality, emergency readmission rates, and Patient Reported Outcome Measures (PROMs).

The risk-adjusted SHMI is the ratio of the number of patients who either died in-hospital or within 30 days after discharge to the number that would be expected to die on the basis of average England figures, given the characteristics of patients treated. The SHMI is an index with baseline at 100, meaning that the trust experienced its observed deaths to exactly match its expected deaths. A SHMI equal to 90 (115) implies that the trust had 10% less (15% more) deaths than expected. SHMI data are also available for selected diagnoses from 2013/14 to 2017/18. In addition to overall mortality, we consider three high-volume emergency conditions: acute cerebrovascular disease (including stroke), acute myocardial infarction (AMI), and hip fracture. We also use the risk-adjusted mortality rate for surgeries following a non-elective admission available for 2010/11–2014/15.^12^

The risk-adjusted emergency readmission rate measures the indirectly standardised percentage of emergency admissions to any hospital in England occurring within 30 days of the last, previous discharge from hospital.^13^ Data are available for 2013/14–2017/18.

The risk-adjusted PROMs evaluate average health gains in patients undergoing primary hip and knee replacements. PROMs compare patient’s self-assessed health status, based on the Oxford Hip and Knee Scores (OHS and OKS, respectively) questionnaires, before surgery and six months after surgery.^14^

We focus on different measures because high bed occupancy can directly affect quality through increases in trolley waiting times and delay admissions in the emergency department. In turn, these can increase staff workloads and lead medical staff to discharge patients prematurely which may increase mortality or readmissions. Moreover, high bed occupancy could also result in longer waiting times for elective procedures and cancellations leading to slower recovery for elective surgeries, as captured by PROMs.

### Independent variables

Hospital bed occupancy rate, our key explanatory variable, is the ratio of occupied to available beds published quarterly in NHS England Statistics. In particular, occupied (available) beds are computed as the average daily number of occupied (available) beds over the quarter. For wards which are open overnight, an occupied bed is defined as one which is occupied at midnight. Given that our quality measures are at the annual level, we average bed occupancy rates across the four quarters.

Our focus is on overnight bed occupancy rates for the general and acute sector.^15^ This indicator allows us to disentangle how overall pressure on beds is associated with quality performance in different areas of acute hospitals. Policymakers and managers will be informed whether high bed occupancy signals lower quality using indicators that cover all treatments (i.e. overall mortality, emergency readmissions) and specific high-volume diagnoses and procedures (i.e. procedure and condition-specific outcomes for heart attack, hip fracture, and stroke). For example, high bed occupancy might not be associated with overall mortality but positively with stroke mortality, therefore policymakers could target policies towards this group of patients.

We include several control variables measured at the hospital level, which can explain the association between bed occupancy and quality. We control for type of hospital: teaching, foundation, and London trust dummies. Hospitals may differ in the availability of doctors, skill mix or non-clinical staff. We measure skill mix (full-time equivalent) with the proportion of doctors to clinical staff and the proportion of managers to total staff.^16^ To control for unavoidable cost differences in labour and capital between hospitals, we include the Market Forces Factor (MFF) based on geographical location published by NHS Improvement. As a proxy of hospital competition, we measure the number of acute hospital trusts located within a 30 km radius from a specific trust. Hospital catchment area is defined as a 15 km radius circle [43, 44].^17^

We also include demographic and socioeconomic variables that capture features of the catchment area. Each hospital is assigned the data from Lower Layer Super Output Areas (LSOA)^18^ whose centroids are located within 15 km from the trust headquarter. These measures consist of the proportion of adults aged 65 and over, population density, proportion of rural LSOA, proportion of non-white individuals, proportion of individuals with a degree, proportion of individuals with a disability, and proportion of income-deprived individuals. Proportion of adults aged 65 and over and population density are computed using annual mid-year population estimates available from the Office for National Statistics. The remaining variables, except for income-deprived individuals, are single snapshots calculated using 2011 Census data. Finally, we use the 2015 Index of Multiple Deprivation to compute the proportion of income-deprived individuals.

We also include hospital beds, LOS, and inpatient admissions (proxy of volume) as the determinants of bed occupancy rates. Hospital beds are measured as available beds averaged across quarters from NHS England Statistics. LOS is the mean of all patients’ spell duration in days, where a spell is a period of continuous admitted patient care within a particular provider calculated by subtracting the admission date from the discharge date (day-cases whose LOS is zero days are excluded). We obtain inpatient admissions by subtracting day-cases from finished admissions episodes, which count those episodes first in the spell of admitted patient care. We also compute the inpatient admissions to beds ratio in line with our theoretical framework.^19^ NHS Digital reports LOS and admission data.

## Results

### Descriptive statistics

Table 1 presents summary statistics. The number of hospital trusts ranges from 135 for surgical mortality to 150 for SHMI.^20^ For SHMI and PROMs’ samples, a trust is observed 7.3 (out of 8) years on average. For other dependent variables, a trust is observed more than 4.7 (out of 5) years on average.

The overall and condition-specific SHMI are about 100 as these are the ratio of actual to expected deaths. Average surgical mortality is 3.67%. Emergency readmission rates are 13.26%. Patients undergoing a hip and knee replacement have an average health gain of 20.84 and 15.76 points in their OHS and OKS, respectively. Low pairwise correlations across almost all quality variables, reported in Table 5 in the Appendix, show that these indicators measure different dimensions of quality.

Descriptive statistics of bed occupancy rates and control variables in Table 1 are calculated for SHMI’s sample. Bed occupancy rate is on average 88.89%.^21^ Doctors account for 22.64% of clinical staff and managers for 2.27% of total staff. MFF is on average 100 by construction and hospital trusts have around seven competitors. 21% are teaching trusts, 58.8% are foundation trusts, and 14.6% are located in London. Hospital trusts have on average 700 beds, 62,300 inpatient admissions per year, 89 inpatients per bed, and patients stay more than 4 days. Correlation between beds and inpatient admissions is 0.921 showing high collinearity (Table 7 in the Appendix). Concerning hospital catchment areas, 17.03% of individuals are aged 65 or over, 14.62% are non-white, 27.7% have a degree, 17.68% have a disability, and 14.76% are income-deprived. The population density is 1,684 individuals per square kilometre on average and 13.69% of LSOA in the catchment area are considered rural.^22^

**Table 1:** Descriptive statistics

Variable	Obs	Trusts	*T*	Mean	Standard deviation			Min	Max
					Overall	Between	Within		
**Dependent variable**									
SHMI	1104	150	7.360	100.2	9.592	8.959	4.452	53.90	124.8
SHMI (stroke)	674	143	4.713	102.1	16.48	14.51	9.682	44.45	169.7
SHMI (AMI)	669	143	4.678	100.3	23.96	18.74	15.68	36.96	211.9
SHMI (hip fracture)	669	142	4.711	102.1	23.46	17.91	16.25	41.09	246.3
Surgical mortality rate (%)	669	135	4.956	3.670	0.717	0.578	0.424	1.858	6.448
Emergency readmission rate (%)	681	143	4.762	13.26	1.247	1.034	0.698	8.900	17.90
Health gain hip replacement	1047	144	7.271	20.84	1.484	0.975	1.173	14.88	24.92
Health gain knee replacement	1054	144	7.319	15.76	1.421	1.081	1.025	6.678	19.78
**Independent variable**									
Bed occupancy rate (%)	1104	150	7.360	88.89	5.168	4.089	3.154	62.69	99.28
**Demand–supply shifters**									
Prop. of doctors (%)	1104	150	7.360	22.64	3.161	2.936	1.202	9.272	38.88
Prop. of managers (%)	1104	150	7.360	2.266	0.816	0.776	0.294	0.409	5.670
Market forces factor	1104	150	7.360	99.63	6.273	6.461	0.294	92.30	120.0
Hospital competition	1104	150	7.360	7.596	8.932	9.535	0.951	0	32
Prop. of indiv. aged 65 + (%)	1104	150	7.360	17.03	3.671	3.730	0.673	9.672	26.72
Population density (1000)	1104	150	7.360	1.684	2.042	2.129	0.074	0.076	8.493
Prop. of rural LSOA (%)	1104	150	7.360	13.69	13.75	13.76	0.000	0.000	63.29
Prop. of non-white indiv. (%)	1104	150	7.360	14.62	13.04	13.72	0.000	1.322	44.23
Prop. of indiv. with degree (%)	1104	150	7.360	27.70	7.199	7.311	0.000	15.31	44.16
Prop. of indiv. with disability (%)	1104	150	7.360	17.68	2.981	3.004	0.000	12.53	24.31
Prop. of income-deprived indiv. (%)	1104	150	7.360	14.76	4.045	4.035	0.000	6.694	24.09
Teaching trust	1104	150	7.360	0.210	0.408	0.395	0.104	0	1
Foundation trust	1104	150	7.360	0.588	0.492	0.486	0.112	0	1
London trust	1104	150	7.360	0.146	0.353	0.380	0.000	0	1
**Determinants of bed occupancy rate**									
Beds (1000)	1104	150	7.360	0.707	0.316	0.327	0.067	0.196	2.025
Length of stay	1104	150	7.360	4.227	0.613	0.617	0.255	2.777	7.600
Admissions (100,000)	1104	150	7.360	1.041	0.468	0.488	0.114	0.232	3.045
Inpatient admissions (100,000)	1104	150	7.360	0.623	0.280	0.290	0.067	0.102	1.867
Inpatients to beds ratio	1104	150	7.360	89.22	14.90	14.57	6.904	43.21	160.0

Descriptive statistics for bed occupancy rate, SHMI, and controls are computed for SHMI’s sample. All other dependent variables are reported for their own sample. SHMI and health gains are published for 2010/11–2017/18. Surgical mortality rates are published for 2010/11–2014/15. SHMI by diagnosis and emergency readmission rates are published for 2013/14–2017/18

*Obs*.  = number of observations, *T =*  average number of years a trust is observed, *Min =*  minimum, *Max =*  maximum, *SHMI =* Summary Hospital-level Mortality Indicator, *AMI =* acute myocardial infarction, *Prop =* proportion, *Indiv =* individuals, *LSOA =* Lower Layer Super Output Areas

### Main results

Table 2 provides our key results for Models 1–6. Models 1–5 report the association between bed occupancy rate and quality, after controlling for different set of controls, and Model 6 decomposes this into the between association (first row) and within association (second row).

**Table 2:** Results for the association between bed occupancy rates and quality

	Model 1	Model 2	Model 3	Model 4	Model 5	Model 6
**SHMI**						
Bed occupancy rate	0.221* [−0.021, 0.463]	0.240*** [0.098, 0.382]	0.242*** [0.102, 0.383]	0.238*** [0.100, 0.376]	0.182** [0.042, 0.322]	0.337*** [0.122, 0.552]
Deviation bed occupancy rate (within association)						−0.047 [−0.153, 0.060]
*R*^2^	0.014	0.506	0.507	0.519	0.517	0.535
**SHMI (stroke)**						
Bed occupancy rate	0.257 [−0.223, 0.736]	0.252 [−0.089, 0.592]	0.267 [−0.081, 0.615]	0.269 [−0.085, 0.623]	0.299* [−0.057, 0.654]	0.519* [−0.049, 1.087]
Deviation bed occupancy rate (within association)						0.042 [−0.356, 0.440]
*R*^2^	0.007	0.195	0.198	0.202	0.199	0.243
**SHMI (AMI)**						
Bed occupancy rate	0.176 [−0.400, 0.752]	0.164 [−0.397, 0.724]	0.181 [−0.366, 0.728]	0.187 [−0.351, 0.724]	0.156 [−0.411, 0.723]	0.053 [−0.708, 0.815]
Deviation bed occupancy rate (within association)						0.244 [−0.389, 0.878]
*R*^2^	0.002	0.099	0.1	0.105	0.1	0.111
**SHMI (hip fracture)**						
Bed occupancy rate	0.327 [−0.181, 0.835]	0.331 [−0.127, 0.789]	0.308 [−0.146, 0.763]	0.308 [−0.147, 0.763]	0.330 [−0.147, 0.807]	0.386 [−0.220, 0.992]
Deviation bed occupancy rate (within association)						0.298 [−0.266, 0.863]
*R*^2^	0.005	0.08	0.083	0.083	0.083	0.122
**Surgical mortality rate**						
Bed occupancy rate	0.023*** [0.006, 0.039]	0.025*** [0.013, 0.038]	0.028*** [0.015, 0.040]	0.027*** [0.015, 0.040]	0.022*** [0.010, 0.035]	0.024*** [0.007, 0.042]
Deviation bed occupancy rate (within association)						0.017*** [0.006, 0.027]
*R*^2^	0.098	0.356	0.367	0.373	0.379	0.395
**Emergency readmission rate**						
Bed occupancy rate	−0.007 [−0.041, 0.027]	−0.009 [−0.036, 0.019]	−0.008 [−0.037, 0.020]	−0.009 [−0.036, 0.018]	0.001 [−0.025, 0.027]	−0.002 [−0.040, 0.036]
Deviation bed occupancy rate (within association)						0.009 [−0.019, 0.037]
*R*^2^	0.054	0.31	0.31	0.32	0.331	0.374
**Health gain hip replacement**						
Bed occupancy rate	−0.021* [−0.043, 0.002]	−0.023** [−0.043, −0.002]	−0.021** [−0.041, −0.000]	−0.021** [−0.041, −0.000]	−0.022** [−0.042, −0.002]	−0.038** [−0.069, −0.007]
Deviation bed occupancy rate (within association)						0.001 [−0.022, 0.024]
*R*^2^	0.369	0.462	0.465	0.465	0.466	0.482
**Health gain knee replacement**						
Bed occupancy rate	−0.027** [−0.053, −0.000]	−0.026** [−0.047, −0.006]	−0.026** [−0.046, −0.006]	−0.026** [−0.047, −0.006]	−0.023** [−0.044, −0.001]	−0.039*** [−0.068, −0.010]
Deviation bed occupancy rate (within association)						−0.001 [−0.021, 0.019]
*R*^2^	0.283	0.471	0.471	0.472	0.473	0.499

Model 1 reports Pooled OLS regression of quality on bed occupancy rates controlling for year fixed effects. Model 2 includes exogenous controls and year fixed effects. Model 3 includes controls in Model 2 and beds. Model 4 (5) includes controls in Model 3 and inpatients to beds ratio (length of stay). Model 6 shows results of the within–between random-effects specification for Model 5 and reports the between association in the row of the overall association for the other models. Controls and year dummies are not reported. Standard errors are clustered at trust level and confidence intervals at 95% level are in brackets. Parameters statistically significant at 1% (***), 5% (**), and 10% (*) levels are reported next to the coefficient. *SHMI* = Summary Hospital-level Mortality Indicator, *AMI* = acute myocardial infarction. SHMI and health gains are published for 2010/11–2017/18. Surgical mortality rates are published for 2010/11–2014/15 and SHMI by diagnosis and emergency readmissions rates are published for 2013/14–2017/18. Total observations are 1,104 for SHMI, 674 for SHMI (Stroke), 669 for SHMI (AMI), SHMI (Hip Fracture), and surgical mortality rates, 681 for emergency readmission rates, 1,047 for average health gain after hip replacement, and 1054 for average health gain after knee replacement

#### Model 1: Are high bed occupancy rates a signal of low quality?

Our results for Model 1 show that higher bed occupancy is positively associated with SHMI (at 10% significance level) and surgical mortality (at 1%) —higher mortality implies lower quality— and negatively associated with average health gain after hip replacement (at 10%) and knee replacement (at 5%), while there is no statistically significant association with condition-specific SHMI and emergency readmissions. Therefore, a regulator can infer that high bed occupancy rates are a signal of low quality for overall and surgical mortality and health gains for elective surgeries and could initiate additional monitoring or auditing to hospitals experiencing high bed occupancy rates.

In more detail, a one standard deviation increase in bed occupancy (5p.p.) is associated with an increase of 1.105p.p. in overall mortality (which corresponds to a 1.1% increase relative to a mean SHMI mortality indicator of 100.2 that measures the ratio of observed deaths over expected deaths), which is one ninth of its standard deviation (1.105/9.592 = 0.12). This means that hospitals with higher bed occupancy by 5p.p. have 1.1% higher deaths, which is equivalent to 685 additional inpatient deaths (mean of 62,300 inpatient admissions) and 1,145 total deaths (mean of 104,100 total admissions) per year.

A one standard deviation increase in bed occupancy is also associated with an increase of 0.115p.p. in surgical mortality (which corresponds to a 3.13% increase relative to a mean surgical mortality rate of 3.67%), which is 0.16 of its standard deviation (= 0.115/0.717). A one standard deviation increase in bed occupancy is associated with a decrease of 0.105 points in health gain after a hip replacement (which corresponds to a 0.5% relative to a mean of 20.84 points in OHS) and 0.135 points after a knee replacement (which corresponds to a 0.86% relative to a mean of 15.76 points in OKS) and account for 0.07 and 0.09 of their standard deviations, respectively.

Alternatively, a 1p.p. increase in bed occupancy rates is associated with an increase of 0.2% in overall mortality and of 0.6% in surgical mortality, and a reduction of 0.1% in health gain after a hip replacement and 0.2% after a knee replacement.

#### Model 2: Do exogenous demand and supply factors explain the association?

In the conceptual framework, we argue that exogenous demand and supply factors might directly and also indirectly affect quality and, therefore, explain the association of interest. If that was the case, regulators could identify clusters of hospitals with similar characteristics that show both high bed occupancy rates and low quality.

Model 2 shows that demand–supply shifters do not explain the associations identified by Model 1. Although some variables are associated with quality, the associations between quality and bed occupancy rates remain mostly unaltered after the inclusion of demand–supply variables, possibly due to the low correlation with bed occupancy rates. In more detail, the associations between bed occupancy and quality are not explained by higher costs (MFF), staff skill mix, competition, type of hospital or demographics. Thus, regulators cannot rely on common demand and supply factors to target hospitals with high bed occupancy and low quality.

The full results including all explanatory variables are in Table 3,^23^^,^^24^ which we briefly comment on. Hospital catchment areas with more deprived populations are associated with higher overall, stroke and hip fracture mortality. Those with a higher proportion of non-white individuals are associated with more readmissions and lower health gains for hip replacement but lower overall mortality. A higher proportion of individuals with a disability are associated with higher readmissions and lower health gains but lower stroke and hip fracture mortality.

**Table 3:** Results for Model 5 including covariates

	SHMI	SHMI (stroke)	SHMI (AMI)	SHMI (hip fracture)	Surgical mort. rate	Emerg. read	Health gain hip repl	Health gain knee repl
Bed occupancy rate	0.182** [0.042,0.322]	0.299* [−0.057,0.654]	0.156 [−0.411,0.723]	0.330 [−0.147,0.807]	0.022*** [0.010,0.035]	0.001 [−0.025,0.027]	−0.022** [−0.042,−0.002]	−0.023** [−0.044,−0.001]
Beds	0.165 [−3.201,3.530]	3.796 [−3.843,11.436]	3.576 [−8.537,15.688]	−5.641 [−17.832,6.550]	0.303** [0.047,0.559]	0.111 [−0.488,0.710]	0.338 [−0.172,0.848]	0.061 [−0.413,0.534]
Length of stay	1.845*** [0.481,3.209]	−1.161 [−4.650,2.329]	0.948 [−4.154,6.050]	−0.769 [−5.252,3.715]	0.150** [0.021,0.279]	−0.352*** [−0.583,−0.121]	0.035 [−0.183,0.253]	−0.121 [−0.311,0.068]
Prop. of doctors	−0.093 [−0.467,0.281]	−0.173 [−1.017,0.671]	0.106 [−0.920,1.131]	−0.392 [−1.435,0.652]	0.000 [−0.027,0.028]	0.024 [−0.034,0.081]	0.004 [−0.042,0.051]	−0.033 [−0.096,0.030]
Prop. of managers	−1.189*** [−2.050,−0.327]	−2.056 [−4.632,0.519]	−3.537* [−7.442,0.369]	−2.881 [−6.791,1.029]	−0.010 [−0.116,0.096]	0.017 [−0.195,0.230]	0.062 [−0.099,0.222]	−0.057 [−0.205,0.091]
Market forces factor	−0.087 [−0.538,0.364]	0.052 [−0.969,1.074]	1.200* [−0.113,2.514]	−1.091* [−2.245,0.063]	0.013 [−0.028,0.054]	−0.008 [−0.084,0.067]	0.008 [−0.063,0.078]	−0.037 [−0.095,0.020]
Hospital competition	0.299** [0.055,0.544]	0.786** [0.177,1.395]	0.830* [−0.066,1.725]	1.420*** [0.497,2.343]	0.022** [0.004,0.041]	−0.033 [−0.082,0.015]	−0.016 [−0.058,0.026]	−0.010 [−0.056,0.036]
Prop. of individuals aged 65 +	0.123 [−0.546,0.793]	1.715** [0.175,3.254]	1.455 [−0.733,3.642]	1.038 [−1.308,3.383]	−0.036 [−0.096,0.024]	−0.182*** [−0.297,−0.066]	0.071 [−0.034,0.176]	0.075 [−0.021,0.172]
Population density	−1.725** [−3.363,−0.087]	−2.870* [−5.907,0.167]	−0.060 [−4.963,4.844]	−0.602 [−4.901,3.698]	−0.037 [−0.182,0.109]	0.134 [−0.143,0.411]	0.151 [−0.094,0.397]	−0.034 [−0.235,0.167]
Prop. of rural LSOA	0.050 [−0.051,0.150]	−0.029 [−0.228,0.170]	0.440*** [0.168,0.711]	0.118 [−0.229,0.466]	0.002 [−0.007,0.011]	−0.011 [−0.028,0.006]	0.002 [−0.011,0.015]	−0.003 [−0.016,0.010]
Prop. of non-white individuals	-0.136** [-0.267,-0.005]	-0.241* [-0.526,0.044]	0.022 [-0.495,0.539]	-0.266 [-0.725,0.192]	-0.001 [-0.015,0.013]	0.033** [0.005,0.060]	-0.035** [-0.065,-0.005]	-0.015 [-0.037,0.007]
Prop. of individuals with degree	−0.364** [−0.714,−0.013]	0.162 [−0.546,0.870]	0.093 [−0.832,1.018]	0.067 [−0.845,0.978]	−0.015 [−0.041,0.011]	0.015 [−0.045,0.074]	0.018 [−0.028,0.065]	−0.016 [−0.058,0.026]
Prop. of individuals with a disability	−0.823* [−1.789,0.143]	−2.524** [−4.849,−0.199]	−1.589 [−5.069,1.890]	−3.742** [−6.897,−0.587]	0.055 [−0.049,0.160]	0.331*** [0.128,0.535]	−0.182** [−0.348,−0.017]	−0.178** [−0.331,−0.025]
Prop. of income-deprived individuals	0.664** [0.052,1.276]	2.026*** [0.539,3.513]	2.614* [−0.173,5.400]	2.118** [0.011,4.224]	0.018 [−0.048,0.084]	−0.105* [−0.227,0.017]	0.034 [−0.069,0.136]	0.032 [−0.065,0.128]
Teaching trust	−3.795*** [−6.059,−1.531]	−1.156 [−7.459,5.147]	7.537 [−1.489,16.563]	5.254 [−2.658,13.167]	0.372** [0.087,0.656]	−0.127 [−0.538,0.284]	−0.053 [−0.404,0.298]	0.001 [−0.347,0.348]
Foundation trust	−0.688 [−2.492,1.117]	−1.075 [−4.921,2.772]	−4.496 [−10.839,1.848]	−4.719 [−10.570,1.133]	−0.030 [−0.169,0.110]	0.140 [−0.142,0.421]	−0.031 [−0.293,0.231]	−0.139 [−0.390,0.111]
London trust	−5.060* [−10.151,0.031]	−16.491** [−29.767,−3.216]	−29.112*** [−47.192,−11.031]	−16.520 [−36.509,3.470]	−0.189 [−0.739,0.360]	−0.147 [−1.431,1.137]	−0.588 [−1.512,0.336]	−0.264 [−1.086,0.557]
Constant	106.443*** [43.888,168.998]	68.851 [−65.391,203.092]	−78.378 [−256.534,99.777]	214.653*** [60.475,368.832]	−0.652 [−5.594,4.289]	12.471** [2.809,22.134]	21.409*** [11.506,31.313]	24.064*** [16.320,31.808]
Observations	1104	674	669	669	669	681	1047	1054
*R*^2^	0.517	0.199	0.100	0.083	0.379	0.331	0.466	0.473

Model 5 reports pooled OLS regression of quality on bed occupancy rates controlling for beds, length of stay, proportion of doctors and managers, market forces factor, hospital competition, proportion of individuals aged 65 and over, population density, proportion of rural LSOA, proportion of non-white individuals, proportion of individuals with a degree, proportion of individuals with a disability, proportion of income-deprived individuals, teaching, foundation, and London dummies, and year fixed effects. Year dummies are not reported. Standard errors are clustered at trust level and confidence intervals at 95% level are in brackets. Parameters statistically significant at 1% (***), 5% (**), and 10% (*) levels are reported next to the coefficient. *SHMI* = Summary Hospital-level Mortality Indicator, *AMI*  = acute myocardial infarction, *Mort* = mortality, *Emerg. Read.* = emergency readmission rate, *Repl* = replacement, *Prop* = proportion, *LSOA* = Lower Layer Super Output Areas. SHMI and health gains are published for 2010/11–2017/18. Surgical mortality rates are published for 2010/11–2014/15 and SHMI by diagnosis and emergency readmissions rates are published for 2013/14–2017/18

On the supply side, hospitals located in London have lower overall, stroke and heart attack mortality possibly due to better equipment and ability to recruit more qualified staff. Hospitals with more competitors are associated with higher overall, stroke, hip fracture and surgical mortality. This is in contrast to previous studies [45, 46], though our results are derived from pooled cross-sectional models, may be subject to omitted variable bias and use recent years relative to the 2006 NHS choice reform exploited in previous studies.

#### Models 3–5: Is the association due to the determinants of bed occupancy rates?

The conceptual framework shows that beds, LOS and volume determine bed occupancy rates and might be associated with quality directly and indirectly. Adding sequentially the three key determinants allows identifying which source of variation in bed occupancy rates is responsible for the association with quality after controlling for exogenous demand and supply shifters.

Models 3 and 4 show that results from Models 1 and 2 are robust to the inclusion of beds and inpatients per bed. This implies that hospital capacity and volume determine bed occupancy rates, but they are not the source of variation explaining the association with quality. Alternatively, Model 5 suggests that the association is mostly due to LOS, except for average health gain after a hip replacement. LOS explains 24.79% of the association with SHMI, 21.43% of the association with surgical mortality, and 11.54% of the association with health gain after a knee replacement (comparing Model 3 with 5).

Table 3 reports that LOS is positively associated with overall and surgical mortality and negatively with health gain after a knee replacement (although not statistically significant), which explains the reduction in bed occupancy coefficient. This is in line with longer stays increasing bed occupancy rates as well as patient exposure to hospital-acquired infections and other adverse events which can negatively impact hospital quality. Interventions in the form of shortening LOS might decrease bed occupancy rates, while alleviating their negative association with important dimensions of quality.

Several mechanisms might explain the remaining association between bed occupancy and quality, after further controlling for LOS. High bed occupancy implies that hospitals are closer to full capacity. Patients might be placed in alternative wards whose staff are less specialised. Staff under pressure may carry out tasks in a hurry, reduce patient attention and face higher stress levels when patient-to-staff ratios are higher [31]. Health outcomes could also be worse if patients had to wait longer [47] before being admitted due to less capacity. Hospitals with high bed occupancy rates might give priority to patients with more urgent conditions, such as a stroke, heart attack or hip fracture, at the expense of less urgent conditions (encompassed in overall and surgical mortality) and elective patients. English hospitals may discharge prematurely low-severity patients who are less likely to need an emergency readmission [12].

#### Model 6: Do variations between hospitals rather than within hospitals explain the association?

Model 6 suggests that it is mostly variations between hospitals that explain the association when this is present, except for surgical mortality where variations within hospitals also play a role. Therefore, regulators can focus on targeting hospitals whose bed occupancy rates are systematically high rather than focusing on hospitals experiencing increases in bed occupancy rates over time.

The time-invariant component of bed occupancy across hospitals could be related to hospitals’ organisational ability and efficiency in the use of their resources, e.g. due to management quality, skills and leadership. Hospitals with worse management could lead to higher bed occupancy as a result of lower organisational ability, but also to worse quality and health outcomes. Variations in organisation and management quality could also vary over time, as hospitals adapt to changing demand characteristics, new policies, environmental trends, budgets, etc., therefore contributing to the association between bed occupancy rates and surgical mortality within hospitals.

### Robustness checks

Tables 9 and 10 in the Appendix show the results for non-linear regressions. The results are broadly in line with the linear regressions. In Models 1 to 4, bed occupancy below 85% (above 90%) is associated with lower (higher) overall and surgical mortality and, therefore, the association is monotonic. This association is mostly explained by LOS as shown in Model 5. For Model 6, neither variations in bed occupancy rates between hospitals nor within hospitals are statistically significant at 5% level, except for the negative between association of bed occupancy with non-elective mortality for the first category (≤ 85%). Health gains are higher when bed occupancy rates are below 85% and lower when above 90%, even though only the 90%–95% band is statistically significant at 1% level in almost all models. There is no significant association between bed occupancy rates and AMI mortality and emergency readmissions. Differently from the linear results, bed occupancy rates above 95% are positively associated with stroke and hip fracture mortality.

Tables 11 in the Appendix shows the results for a balanced panel. Bed occupancy is positively associated with SHMI in Model 1. Again, the association becomes stronger in Model 2 and is mainly explained by LOS (Model 5) and variations across hospitals (Model 6). Similar conclusions are derived from the results for condition-specific SHMI (SHMI stroke has significant coefficients but only at the 10% level), surgical mortality, and emergency readmissions. The association with PROMs for hip and knee replacements is not statistically significant for Model 1, but the results are fairly robust for the remaining models.

## Conclusion

We have investigated whether hospitals with high bed occupancy rates are associated with lower quality and the factors explaining such association in the English NHS in 2010–2018. Our results show that higher bed occupancy is negatively associated with some quality indicators (overall and surgical mortality, and health gains), while there is no association with condition-specific mortality and emergency readmissions. A 5p.p. increase in bed occupancy is associated with an increase of 1.1% in overall mortality and of 3.1% in surgical mortality and a reduction of 0.5% and 0.9% in health gain for hip and knee replacement, respectively. Therefore, although the association is only present for a subset of indicators, when detected it appears quantitatively important. For example, the overall mortality effect is equivalent to 685 additional inpatient deaths. We focus on a 5p.p. increase as this is equivalent to a standard deviation in bed occupancy rates that we observe in the data. It could be argued that this is a large increase in bed occupancy rates and that individual providers could realistically change their bed occupancy rates in the order of one or two p.p., in which case the effects would be one or two fifths of those outlined above.

Our analysis suggests that 12%–25% of the association is explained by patients’ LOS and the remaining by variations in bed occupancy between hospitals. We do not find that demand–supply factors, beds, and volume have a significant role in explaining such associations.

Our results are in line with the positive association between bed occupancy and overall mortality found in other studies [1, 11, 14, 15]. Our estimate of 1.1% increase in overall mortality for 5p.p. increase in bed occupancy lies between the 4.5% and 4.8% increase for 3p.p. increase in bed occupancy estimated in Boden et al. [11] and the 1.2% for 10% rise in bed occupancy in Madsen et al. [14]. We find no association with emergency readmissions similar to Friebel et al. [12] and contrary to Blom et al. [10].

These findings have policy implications. High bed occupancy rates are a signal of lower quality at least for some quality dimensions, and policymakers could monitor or audit those hospitals with high bed occupancy to improve quality of care. Given that demand and supply factors do not explain these associations, there is limited scope for regulators to cluster groups of hospitals based on the population characteristics they serve or hospital characteristics. Instead, high LOS explains a significant portion of the association of quality and bed occupancy rates and therefore LOS can be used as a marker of poorer quality as well. This is potentially an interesting finding because LOS can be generally measured at a more disaggregated level (e.g. by treatment or specialty), relative to bed occupancy rates, and this information could be used by regulators for more targeted interventions. Finally, our results suggest that the association is explained by variations in bed occupancy rates between hospitals. Regulators, therefore, could target hospitals that systematically have high bed occupancy rather than hospitals with sharp increases in bed occupancy rates over time.

Overall, our study has provided a theoretical and empirical framework that shows how regression analysis can support interventions that regulate bed occupancy rates. The main strengths include the use of a wide set of quality measures and a range of control variables to explain the association between quality and bed occupancy rates within and between hospitals. Our study has also some limitations. Most of our quality measures refer to extreme health outcomes, such as mortality or readmissions. We also use patient-reported health outcomes but only for hip and knee replacements, which we interpret as marker conditions and therefore, the results cannot be generalised to other surgeries. Future work could investigate more refined health outcome measures for other treatments as well as going beyond clinical measures of hospital quality such as measures of patient satisfaction. Another limitation is that we have used a limited set of demand–supply determinants and additional determinants (e.g. hospital management, staff stress), which could be the focus of future research as data become available. Last, our analysis relies on hospital quality measures at the hospital level which are already risk adjusted. Future work could explore the role of different risk adjustment models.

## Data Availability

The authors declare that the data used in this article is in the public domain.

## Appendix

See Tables 4, 5, 6, 7, 8, 9, 10, 11 and 12.

**Table 4:** Definition and online links of data

Variable	Definition and link
Bed occupancy rate	Bed occupancy rate is the ratio of average daily number of occupied beds to average daily number of available beds. The analysis focuses on general and acute overnight bed occupancy rates. Overnight and day-case (occupied and available) beds are reported. For wards open overnight, an occupied bed is defined as one which is occupied at midnight on the day in question. For wards open day only, an occupied bed is defined as a bed in which at least one day-case has taken place during the day. Although it is common practice for day-case beds to be used by more than one patient during a day, for wards open day only an occupied bed is defined as a bed in which at least one day-case has taken place during the day. The number of overnight and open day-case beds do not overlap since the methodology followed in the calculation distinguishes between wards open overnight and wards open day only. The variable only includes beds in units managed by the provider and excludes beds commissioned from other providers. The following beds are excluded: available and occupied beds designated solely for the use of well babies, critical care beds, residential care beds, and beds of patients under non consultant-led care, i.e. nurse/therapy or GP led. A bed allocated to a patient on home leave is recorded as not available and therefore not occupied. Data are published quarterly Source: NHS England Statistics https://www.england.nhs.uk/statistics/statistical-work-areas/bed-availability-and-occupancy/
Summary Hospital-level Mortality Indicator	The risk-adjusted SHMI is the ratio of the actual number of patients who died following hospitalisation at the trust to the number that would be expected to die on the basis of average England figures, given the characteristics of the patients treated there. The numerator of this ratio includes all deaths reported of patients who were admitted and either died while in-hospital or within 30 days of discharge. If the patient is treated by another trust within 30 days after discharge, their death is only attributed to the last trust to treat them. The expected deaths are estimated through a logistic regression controlling for age, gender, admission method, year index, Charlson Comorbidity Index, and diagnosis grouping. A three-year dataset is used to create this risk-adjusted model. The SHMI is composed of 140 different diagnosis groups aggregated to calculate the overall SHMI. From 2013/14, the SHMI data has been also published by diagnosis group. The diagnosis included in the study are: acute cerebrovascular diseases (ICD-10 codes G46.0 to G46.8, I60.0 to I63.9 -except I61.7, I62.2 to I62.8, I63.7-, I64.X, I66.0 to I66.4, I66.8, I66.9), acute myocardial infarction (ICD-10 codes I21.0 to I21.4, I21.9 to I22.1, I22.8, I22.9), and hip fracture (ICD-10 codes S72.0, S72.1, S72.2). Data are published annually Source: NHS Digital https://digital.nhs.uk/data-and-information/publications/clinical-indicators/shmi
Surgical mortality rate	The risk-adjusted mortality data measures indirectly standardised rates for patients whose death occurred either in-hospital or within 30 days of an operative procedure. The indirectly standardised rate is the ratio between hospital’s observed and expected deaths multiplied by an overall event rate of patients in England. The expected events are the product between the number of patients for a provider and the overall event rate for each risk adjustment category (gender-age combination) summed over all categories. The procedures are surgeries following a non-elective admission (patients with diagnosis of cancer are excluded). All aged patients are considered. Data are available annually up to 2014/15 Source: NHS Digital https://digital.nhs.uk/data-and-information/publications/statistical/compendium-hospital-care/current/deaths-within-30-days
Emergency readmission rate	The risk-adjusted emergency readmission rate measures the indirectly standardised percentage of emergency admissions to any hospital in England occurring within 30 days of the last, previous discharge from hospital. It is calculated as the ratio of the provider’s observed number of readmissions to the number of events that would be expected if it had experienced the same event rates as those of patients in England in the standard population and across the mid-point time period (2015/16), given the case-mix of age, sex, method of admission and diagnosis/procedure of its patients. The expected events are the product between the number of patients for a provider and a crude rate in the standard population for each case-mix group summed over all groups. Then, this standardised ratio is converted into a rate multiplying it by the overall event rate of patients in England. Emergency readmission rates for all conditions are considered. Admissions for cancer and obstetrics are excluded as they may be part of the patient´s care plan. All patients aged 16 and over are included. The data are available annually from 2013/14 to 2017/18 Source: NHS Digital https://digital.nhs.uk/data-and-information/publications/statistical/compendium-emergency-readmissions/current/emergency-readmissions-to-hospital-within-30-days-of-discharge
Patient Reported Outcome Measures	PROMs measure the risk-adjusted average health gain in patients undergoing primary hip and knee replacements in England. PROMs comprise a pair of questionnaires completed by the patient, before and after surgery (at least six months after for hip and knee replacements). The health gain is based on the Oxford Hip and Knee Scores (OHS and OKS, respectively) questionnaires. The surveys include twelve questions related to patient’s pain and mobility, with five multiple choice answers where 0 denotes greatest severity and 4 least or no symptoms. These answers are then summed up to a single score with 0 indicating the worst possible score and 48 the highest possible. OHS is adjusted for age, sex, ethnicity, index of multiple deprivation, pre-operative self-assessed health status, comorbidity, patient assistance to complete the questionnaires, living arrangements, disability, primary diagnosis, and years of experiencing symptoms. OKS have the same adjustment, except for living arrangements and years of experiencing symptoms. Data are published annually Source: NHS Digital https://digital.nhs.uk/data-and-information/publications/statistical/patient-reported-outcome-measures-proms
Control variables	Workforce Statistics Source: NHS Digital https://digital.nhs.uk/data-and-information/publications/statistical/nhs-workforce-statistics Market Forces Factor Source: NHS Improvement https://webarchive.nationalarchives.gov.uk/ukgwa/20200501111106/https://improvement.nhs.uk/resources/reference-costs/ Hospital Competition Source: NHS Digital, Open Geography portal https://data.england.nhs.uk/dataset/ods-nhs-trusts-and-sites https://geoportal.statistics.gov.uk/datasets/ons-postcode-directory-may-2019 Demographic and Socioeconomic Variables Source: Open Geography portal, Office for National Statistics, GOV.UK https://data.gov.uk/dataset/4006b92e-a08d-4e41-addd-2db9c0adeecb/lower-layer-soa-with-names-geometric-centroid-population-weighted-centroid-lookup-table https://www.ons.gov.uk/peoplepopulationandcommunity/populationandmigration/populationestimates http://geoportal.statistics.gov.uk/datasets/rural-urban-classification-2011-of-lower-layer-super-output-areas-in-england-and-wales https://www.nomisweb.co.uk/census/2011 https://www.gov.uk/government/statistics/english-indices-of-deprivation-2015 Type of Hospital Source: NHS Digital, NHS England https://digital.nhs.uk/data-and-information/publications/statistical/estates-returns-information-collection https://www.england.nhs.uk/publication/nhs-provider-directory/ Beds Source: NHS England Statistics https://www.england.nhs.uk/statistics/statistical-work-areas/bed-availability-and-occupancy/ Day-Cases, Length of Stay, and Admissions Source: NHS Digital https://digital.nhs.uk/data-and-information/publications/statistical/hospital-admitted-patient-care-activity

**Table 5:** Pairwise correlations across quality measures

	(1)	(2)	(3)	(4)	(5)	(6)	(7)	(8)
(1) SHMI	1							
(2) SHMI (stroke)	0.444*	1						
(3) SHMI (AMI)	0.103*	0.111*	1					
(4) SHMI (hip fracture)	0.250*	0.171*	0.144*	1				
(5) Surgical mortality rate	0.0935*	0.0649	0.183*	0.213*	1			
(6) Emergency readmission rate	−0.188*	−0.0840*	0.0761*	0.108*	0.169*	1		
(7) Health gain hip replacement	−0.0273	−0.0769	0.00945	−0.0773	−0.263*	−0.169*	1	
(8) Health gain knee replacement	0.231*	0.0727	−0.153*	−0.0326	−0.239*	−0.206*	0.541*	1

Correlations statistically significant at the 5% (*) level

*SHMI* = Summary Hospital-level Mortality Indicator, *AMI* = acute myocardial infarction

**Table 6:** Frequencies and number of observations by bed occupancy rate category

	BOR mean	BOR median	Obs	BOR ≤ 85%		85% < BOR ≤ 90%		90% < BOR ≤ 95%		BOR > 95%	
				Obs	Freq	Obs	Freq	Obs	Freq	Obs	Freq
SHMI	88.89%	89.37%	1,104	227	20.56%	379	34.33%	391	35.42%	107	9.69%
SHMI (stroke)	89.58%	89.96%	674	108	16.02%	231	34.27%	256	37.98%	79	11.72%
SHMI (AMI)	89.63%	90.03%	669	106	15.84%	228	34.08%	255	38.12%	80	11.96%
SHMI (hip fracture)	89.57%	89.93%	669	109	16.29%	229	34.23%	251	37.52%	80	11.96%
Surgical mortality rate	88.06%	88.50%	669	172	25.71%	234	34.98%	214	31.99%	49	7.32%
Emergency readmission rate	89.58%	89.93%	681	110	16.15%	234	34.36%	255	37.44%	82	12.04%
Health gain hip replacement	88.79%	89.32%	1,047	221	21.11%	360	34.38%	370	35.34%	96	9.17%
Health gain knee replacement	88.85%	89.35%	1,054	218	20.68%	364	34.54%	373	35.39%	99	9.39%

*BOR* = bed occupancy rate, *SHMI* = Summary Hospital-level Mortality Indicator, *AMI* = acute myocardial infarction, *Obs*. = observations, *Freq*. = frequency

**Table 7:** Correlations across bed occupancy rate and control variables

	(1)	(2)	(3)	(4)	(5)	(6)	(7)	(8)	(9)
(1) Bed occupancy rate	1								
(2) Beds	−0.039	1							
(3) Length of stay	0.170*	0.130*	1						
(4) Inpatient admissions	0.009	0.921*	−0.150*	1					
(5) Inpatients to beds ratio	0.098*	−0.158*	−0.777*	0.187*	1				
(6) Prop. of doctors	0.138*	0.070*	−0.198*	0.147*	0.249*	1			
(7) Prop. of managers	−0.026	−0.189*	0.005	−0.186*	0.036	0.150*	1		
(8) Market forces factor	0.081*	−0.050	-0.103*	0.038	0.281*	0.491*	0.282*	1	
(9) Hospital competition	0.043	−0.030	-0.062*	0.034	0.217*	0.324*	0.192*	0.809*	1
(10) Prop. of individuals aged 65 +	−0.062*	−0.213*	0.093*	−0.283*	−0.247*	−0.359*	−0.157*	−0.725*	−0.752*
(11) Population density	−0.014	0.119*	0.001	0.177*	0.199*	0.269*	0.194*	0.778*	0.883*
(12) Prop. of rural LSOA	−0.091*	−0.249*	−0.063*	−0.258*	−0.055	−0.072*	0.026	−0.392*	−0.578*
(13) Prop. of non-white individuals	0.067*	0.136*	−0.102*	0.235*	0.289*	0.343*	0.150*	0.783*	0.850*
(14) Prop. of individuals with degree	0.028	−0.032	−0.038	0.027	0.181*	0.355*	0.169*	0.785*	0.632*
(15) Prop. of individuals with disability	−0.127*	0.023	0.174*	−0.079*	−0.308*	−0.483*	−0.188*	−0.782*	−0.468*
(16) Prop. of income-deprived individuals	−0.082*	0.237*	0.106*	0.210*	−0.055	−0.160*	−0.037	−0.088*	0.299*
(17) Teaching trust	−0.024	0.528*	0.245*	0.466*	−0.097*	0.174*	−0.010	0.161*	0.174*
(18) Foundation trust	−0.190*	−0.072*	−0.086*	−0.090*	−0.031	−0.163*	−0.060*	−0.151*	−0.170*
(19) London trust	0.025	−0.001	−0.070*	0.067*	0.241*	0.354*	0.188*	0.826*	0.881*

	(10)	(11)	(12)	(13)	(14)	(15)	(16)	(17)	(18)	(19)
(1) Bed occupancy rate										
(2) Beds										
(3) Length of stay										
(4) Inpatient admissions										
(5) Inpatients to beds ratio										
(6) Prop. of doctors										
(7) Prop. of managers										
(8) Market forces factor										
(9) Hospital competition										
(10) Prop. of individuals aged 65 +	1									
(11) Population density	−0.758*	1								
(12) Prop. of rural LSOA	0.595*	−0.584*	1							
(13) Prop. of non-white individuals	−0.840*	0.846*	−0.569*	1						
(14) Prop. of individuals with degree	−0.515*	0.618*	−0.134*	0.599*	1					
(15) Prop. of individuals with disability	0.600*	−0.416*	0.057	−0.593*	−0.796*	1				
(16) Prop. of income-deprived individuals	−0.311*	0.408*	−0.588*	0.285*	−0.371*	0.502*	1			
(17) Teaching trust	−0.310*	0.338*	−0.250*	0.256*	0.221*	−0.119*	0.194*	1		
(18) Foundation trust	0.121*	−0.109*	0.061*	−0.180*	−0.110*	0.134*	0.005	0.097*	1	
(19) London trust	−0.685*	0.886*	−0.399*	0.810*	0.716*	−0.541*	0.175*	0.179*	−0.175*	1

Correlations statistically significant at the 5% (*) level

*Prop*. = proportion, *LSOA* = Lower Layer Super Output Areas

**Table 8:** Dependent and independent variable standard deviations

Variable	Mean	Standard deviation			Within/overall	Within/between
		Overall	Between	Within		
**SHMI's sample**						
Bed occupancy rate	88.893	5.168	4.089	3.154	0.610	0.771
SHMI	100.212	9.591	8.959	4.452	0.464	0.497
**SHMI stroke's sample**						
Bed occupancy rate	89.582	4.918	4.205	2.470	0.502	0.587
SHMI (stroke)	102.128	16.479	14.512	9.682	0.588	0.667
**SHMI AMI's sample**						
Bed occupancy rate	89.634	4.896	4.212	2.397	0.490	0.569
SHMI (AMI)	100.314	23.961	18.740	15.683	0.655	0.837
**SHMI hip fracture's sample**						
Bed occupancy rate	89.569	4.922	4.228	2.478	0.503	0.586
SHMI (hip fracture)	102.084	23.455	17.906	16.252	0.693	0.908
**Surgical mortality's sample**						
Bed occupancy rate	88.057	5.348	4.487	2.969	0.555	0.662
surgical mortality rate	3.670	0.717	0.578	0.424	0.591	0.734
**Emerg. readmission rates' sample**						
Bed occupancy rate	89.581	4.923	4.219	2.478	0.503	0.587
Emergency readmission rates	13.256	1.247	1.034	0.698	0.560	0.675
**Health gain hip repl.'s sample**						
Bed occupancy rate	88.789	5.187	4.155	3.121	0.602	0.751
Health gain hip replacement	20.842	1.484	0.975	1.173	0.790	1.203
**Health gain knee repl.'s sample**						
Bed occupancy rate	88.854	5.168	4.107	3.095	0.599	0.754
Health gain knee replacement	15.760	1.421	1.081	1.025	0.721	0.948

*SHMI* = Summary Hospital-level Mortality Indicator, *AMI* = acute myocardial infarction, *Emerg*. = emergency, *Repl*. = replacement

Table 8 reports the overall (across hospitals and time), the between (across hospitals), and the within (across time) standard deviations for bed occupancy rates and hospital quality for each quality measure’s sample. The within variation is more than half the overall variation and the between variation for bed occupancy rates and quality measures (fifth and sixth columns), except for SHMI. However, the within variation is smaller for hospital characteristics and catchment area measures as shown in Table 1

**Table 9:** Non-linear results for summary hospital-level mortality data

		Model 1	Model 2	Model 3	Model 4	Model 5	Model 6
SHMI	BOR ≤ 85%	−2.203* (1.320)	−1.803** (0.903)	−1.843** (0.885)	−1.884** (0.864)	−1.543* (0.877)	−0.228 (0.724)
	90% < BOR ≤ 95%	0.749 (0.904)	1.574** (0.644)	1.572** (0.645)	1.499** (0.630)	1.208* (0.621)	0.171 (0.498)
	BOR > 95%	1.608 (1.379)	2.178** (0.973)	2.206** (0.984)	2.069** (0.999)	1.568 (0.994)	−0.089 (0.786)
	Deviation BOR ≤ 85% (within association)						−3.311 (2.278)
	Deviation 90% < BOR ≤ 95% (within association)						1.667 (1.606)
	Deviation BOR > 95% (within association)						3.024 (2.004)
	*R*^2^	0.016	0.509	0.510	0.522	0.519	0.538
SHMI (stroke)	BOR ≤ 85%	−2.419 (3.234)	−1.142 (2.272)	−1.412 (2.261)	−1.557 (2.280)	−1.516 (2.241)	−1.642 (2.221)
	90% < BOR ≤ 95%	−0.723 (1.983)	0.561 (1.852)	0.516 (1.860)	0.458 (1.855)	0.751 (1.888)	−0.245 (2.083)
	BOR > 95%	4.964** (2.451)	4.653** (2.296)	4.887** (2.266)	4.737** (2.329)	5.325** (2.428)	2.128 (2.514)
	Deviation BOR ≤ 85% (within association)						−0.206 (4.714)
	Deviation 90% < BOR ≤ 95% (within association)						2.988 (4.006)
	Deviation BOR > 95% (within association)						10.326*** (3.962)
	*R*^2^	0.016	0.199	0.202	0.205	0.203	0.249
SHMI (AMI)	BOR ≤ 85%	−1.575 (3.200)	−2.653 (3.211)	−2.895 (3.204)	−3.112 (3.181)	−2.820 (3.238)	−4.988 (3.444)
	90% < BOR ≤ 95%	1.057 (2.812)	1.014 (2.630)	0.995 (2.631)	0.928 (2.623)	0.844 (2.587)	0.524 (2.199)
	BOR > 95%	3.145 (4.024)	1.055 (3.509)	1.305 (3.501)	1.084 (3.485)	1.022 (3.668)	1.004 (3.546)
	Deviation BOR ≤ 85% (within association)						−0.240 (7.065)
	Deviation 90% < BOR ≤ 95% (within association)						1.105 (4.730)
	Deviation BOR > 95% (within association)						0.620 (6.740)
	*R*^2^	0.004	0.100	0.101	0.106	0.102	0.113
SHMI (hip fracture)	BOR ≤ 85%	2.551 (3.501)	4.116 (3.098)	4.427 (3.042)	4.479 (3.064)	4.311 (3.051)	0.172 (2.968)
	90% < BOR ≤ 95%	3.109 (2.593)	4.009 (2.446)	4.057* (2.445)	4.076* (2.439)	4.274* (2.481)	3.059 (2.780)
	BOR > 95%	10.346*** (3.354)	10.963*** (3.177)	10.628*** (3.235)	10.689*** (3.199)	11.011*** (3.256)	5.869* (3.508)
	Deviation BOR ≤ 85% (within association)						11.619* (6.477)
	Deviation 90% < BOR ≤ 95% (Within association)						7.872* (4.263)
	Deviation BOR > 95% (within association)						16.815*** (5.180)
	*R*^2^	0.018	0.094	0.097	0.097	0.098	0.144

Bed occupancy rates (BOR) is a vector of four categories: ≤ 85%, 85%–90% (baseline), 90%–95%, and > 95%. Model 1 reports Pooled OLS regression of quality on bed occupancy rates controlling for year fixed effects. Model 2 includes exogenous controls and year fixed effects. Model 3 includes controls in Model 2 and beds. Model 4 (5) includes controls in Model 3 and inpatients to beds ratio (length of stay). Model 6 shows results of the within–between random-effects specification for Model 5 and reports the between association in the raw of the overall association for the other models. Controls and year dummies are not reported. Standard errors are clustered at trust level and are in parentheses. Parameters statistically significant at 1% (***), 5% (**), and 10% (*) levels are reported next to the coefficient. *SHMI* = Summary Hospital-level Mortality Indicator, *AMI* = acute myocardial infarction. SHMI is published for 2010/11–2017/18 and SHMI by diagnosis are published for 2013/14–2017/18. Total observations are 1104 for SHMI, 674 for SHMI (stroke), and 669 for SHMI (AMI) and SHMI (hip fracture)

**Table 10:** Non-linear results for surgical mortality rate, emergency readmission rate and average health gains

		Model 1	Model 2	Model 3	Model 4	Model 5	Model 6
Surgical mortality rate	BOR ≤ 85%	−0.131 (0.081)	−0.154** (0.064)	−0.190*** (0.065)	−0.190*** (0.065)	−0.162** (0.065)	0.026 (0.056)
	90% < BOR ≤ 95%	0.164** (0.082)	0.143** (0.069)	0.142** (0.068)	0.137** (0.068)	0.105 (0.069)	0.100* (0.058)
	BOR > 95%	0.229 (0.141)	0.220** (0.095)	0.238** (0.098)	0.231** (0.094)	0.181** (0.091)	0.171* (0.093)
	Deviation BOR ≤ 85% (within association)						−0.408*** (0.154)
	Deviation 90% < BOR ≤ 95% (within association)						−0.005 (0.173)
	Deviation BOR > 95% (within association)						0.167 (0.219)
	*R*^2^	0.099	0.352	0.364	0.370	0.376	0.403
Emergency readmission rate	BOR ≤ 85%	0.051 (0.212)	0.079 (0.171)	0.075 (0.176)	0.091 (0.168)	0.041 (0.163)	−0.106 (0.117)
	90% < BOR ≤ 95%	0.056 (0.147)	−0.022 (0.131)	−0.023 (0.131)	−0.015 (0.129)	0.041 (0.127)	−0.158 (0.105)
	BOR > 95%	−0.208 (0.201)	−0.136 (0.173)	−0.133 (0.173)	−0.119 (0.170)	−0.020 (0.163)	−0.050 (0.179)
	Deviation BOR ≤ 85% (within association)						0.476 (0.353)
	Deviation 90% < BOR ≤ 95% (within association)						0.411* (0.242)
	Deviation BOR > 95% (within association)						−0.045 (0.269)
	*R*^2^	0.058	0.310	0.311	0.320	0.332	0.384
Average health gain hip replacement	BOR ≤ 85%	0.217* (0.124)	0.160 (0.113)	0.134 (0.108)	0.133 (0.108)	0.144 (0.110)	0.123 (0.104)
	90% < BOR ≤ 95%	−0.236* (0.122)	−0.274*** (0.103)	−0.274*** (0.103)	−0.274*** (0.103)	−0.285*** (0.101)	−0.094 (0.094)
	BOR > 95%	−0.012 (0.168)	−0.153 (0.170)	–0.137 (0.174)	−0.135 (0.175)	−0.149 (0.175)	0.043 (0.158)
	Deviation BOR ≤ 85% (within association)						0.239 (0.290)
	Deviation 90% < BOR ≤ 95% (within association)						−0.359 (0.251)
	Deviation BOR > 95% (within association)						−0.252 (0.375)
	*R*^2^	0.376	0.468	0.471	0.471	0.472	0.486
Average health gain knee replacement	BOR ≤ 85%	0.085 (0.145)	0.128 (0.131)	0.123 (0.125)	0.125 (0.126)	0.107 (0.126)	−0.013 (0.109)
	90% < BOR ≤ 95%	−0.368*** (0.119)	−0.321*** (0.097)	−0.321*** (0.097)	−0.320*** (0.097)	−0.303*** (0.099)	−0.043 (0.095)
	BOR > 95%	−0.255 (0.179)	−0.256* (0.154)	−0.254 (0.157)	−0.258 (0.158)	−0.233 (0.161)	−0.023 (0.156)
	Deviation BOR ≤ 85% (within association)						0.239 (0.339)
	Deviation 90% < BOR ≤ 95% (within association)						−0.552** (0.257)
	Deviation BOR > 95% (within association)						−0.171 (0.366)
	*R*^2^	0.292	0.478	0.478	0.478	0.479	0.509

Bed occupancy rates (BOR) is a vector of four categories: ≤ 85%, 85%–90% (baseline), 90%–95%, and > 95%. Model 1 reports Pooled OLS regression of quality on bed occupancy rates controlling for year fixed effects. Model 2 includes exogenous controls and year fixed effects. Model 3 includes controls in Model 2 and beds. Model 4 (5) includes controls in Model 3 and inpatients to beds ratio (length of stay). Model 6 shows results of the within–between random-effects specification for Model 5 and reports the between association in the raw of the overall association for the other models. Controls and year dummies are not reported. Standard errors are clustered at trust level and are in parentheses. Parameters statistically significant at 1% (***), 5% (**), and 10% (*) levels are reported next to the coefficient. Surgical mortality rates are published for 2010/11–2014/15, emergency readmissions rates are published for 2013/14–2017/18, and health gains are published for 2010/11–2017/18. Total observations are 669 for surgical mortality rates, 681 for emergency readmission rates, 1047 for average health gain after hip replacement, and 1054 for average health gain after knee replacement

**Table 11:** Results for the association between bed occupancy rates and quality (balanced panel)

		Model 1	Model 2	Model 3	Model 4	Model 5	Model 6
SHMI	Bed occupancy rate	0.247* (0.130)	0.181** (0.075)	0.193** (0.074)	0.165** (0.068)	0.103 (0.072)	0.226* (0.124)
	Deviation bed occupancy rate (within association)						−0.073 (0.056)
	*R*^2^	0.017	0.502	0.503	0.523	0.517	0.532
SHMI (stroke)	Bed occupancy rate	0.265 (0.257)	0.264 (0.178)	0.330* (0.189)	0.325* (0.192)	0.360* (0.193)	0.447 (0.279)
	Deviation bed occupancy rate (within association)						0.078 (0.212)
	*R*^2^	0.007	0.243	0.257	0.259	0.258	0.282
SHMI (AMI)	Bed occupancy rate	0.201 (0.304)	0.203 (0.304)	0.241 (0.296)	0.231 (0.293)	0.233 (0.314)	0.188 (0.433)
	Deviation bed occupancy rate (within association)						0.292 (0.325)
	*R*^2^	0.005	0.104	0.106	0.108	0.106	0.117
SHMI (hip fracture)	Bed occupancy rate	0.297 (0.264)	0.333 (0.237)	0.276 (0.231)	0.272 (0.232)	0.314 (0.252)	0.348 (0.323)
	Deviation bed occupancy rate (within association)						0.366 (0.295)
	*R*^2^	0.004	0.074	0.078	0.079	0.079	0.106
Surgical mortality rate	Bed occupancy rate	0.023*** (0.009)	0.025*** (0.007)	0.028*** (0.007)	0.027*** (0.006)	0.022*** (0.007)	0.025** (0.010)
	Deviation bed occupancy rate (within association)						0.015*** (0.005)
	*R*^2^	0.098	0.356	0.370	0.377	0.383	0.396
Emergency readmission rate	Bed occupancy rate	−0.011 (0.018)	−0.007 (0.014)	−0.006 (0.015)	−0.007 (0.014)	0.003 (0.014)	−0.002 (0.020)
	Deviation bed occupancy rate (within association)						0.009 (0.015)
	*R*^2^	0.057	0.313	0.314	0.325	0.336	0.375
Health gain hip replacement	Bed occupancy rate	−0.016 (0.012)	−0.024** (0.011)	−0.021* (0.011)	−0.021* (0.011)	−0.021* (0.011)	−0.030* (0.017)
	Deviation bed occupancy rate (within association)						−0.005 (0.013)
	*R*^2^	0.392	0.489	0.492	0.492	0.492	0.500
Health gain knee replacement	Bed occupancy rate	−0.019 (0.014)	−0.026** (0.011)	−0.024** (0.011)	−0.024** (0.011)	−0.019* (0.011)	−0.032** (0.016)
	Deviation bed occupancy rate (within association)						0.006 (0.011)
	*R*^2^	0.291	0.453	0.454	0.454	0.458	0.468

Results for balanced panel. Model 1 reports Pooled OLS regression of quality on bed occupancy rates controlling for year fixed effects. Model 2 includes exogenous controls and year fixed effects. Model 3 includes controls in Model 2 and beds. Model 4 (5) includes controls in Model 3 and inpatients to beds ratio (length of stay). Model 6 shows results of the within–between random-effects specification for Model 5 and reports the between association in the row of the overall association for the other models. Controls and year dummies are not reported. Standard errors are clustered at trust level and are in parentheses. Parameters statistically significant at 1% (***), 5% (**), and 10% (*) levels are reported next to the coefficient. *SHMI* = Summary Hospital-level Mortality Indicator, *AMI* = acute myocardial infarction. SHMI and health gains are published for 2010/11–2017/18. Surgical mortality rates are published for 2010/11–2014/15 and SHMI by diagnosis and emergency readmissions rates are published for 2013/14–2017/18. Total observations are 984 for SHMI, 620 for SHMI (stroke) and SHMI (AMI), 610 for SHMI (hip fracture), 655 for surgical mortality rates, 650 for emergency readmission rates, 888 for average health gain after hip replacement, and 896 for average health gain after knee replacement

**Table 12:** Comparison of within–between random-effects and fixed effects models

	SHMI		SHMI (stroke)		SHMI (AMI)		SHMI (hip fracture)	
	Model 6	FE	Model 6	FE	Model 6	FE	Model 6	FE
Within association	−0.047 (0.054)	−0.047 (0.054)	0.042 (0.203)	0.042 (0.200)	0.244 (0.323)	0.244 (0.318)	0.298 (0.288)	0.298 (0.283)
Observations	1104		674		669		669	
*R*^2^	0.535	0.029	0.243	0.041	0.111	0.021	0.122	0.048

	Surgical mort. rate		Emerg. readmission		Health gain hip repl		Health gain knee repl	
	Model 6	FE	Model 6	FE	Model 6	FE	Model 6	FE
Within Association	0.017*** (0.005)	0.017*** (0.005)	0.009 (0.014)	0.009 (0.014)	0.001 (0.012)	0.001 (0.012)	−0.001 (0.010)	−0.001 (0.010)
Observations	669		681		1047		1054	
R^2^	0.395	0.247	0.374	0.335	0.482	0.561	0.499	0.489

Model 6 shows the within association of the within–between random-effects specification in Eq. (4). FE reports the within association for a hospital fixed effects model. Controls are not reported. Standard errors are clustered at trust level and are in parentheses. Parameters statistically significant at 1% (***), 5% (**), and 10% (*) levels are reported next to the coefficient. *SHMI* = Summary Hospital-level Mortality Indicator, *AMI* = acute myocardial infarction, *Mort* = mortality, *Emerg.* = emergency, *Repl* = replacement, *FE* = fixed effects. SHMI and health gains are published for 2010/11–2017/18. Surgical mortality rates are published for 2010/11–2014/15 and SHMI by diagnosis and emergency readmissions rates are published for 2013/14–2017/18

## Footnotes

1 In the United Kingdom, annual growth rate of government-financed health expenditure in real terms (2018 prices) and adjusted by inflation decreased from 6.1% in 2009 to 1% in 2010, reaching a negative growth of 0.6% in 2013 [48].

2 Data from NHS England Statistics (https://www.england.nhs.uk/statistics/statistical-work-areas/bed-availability-and-occupancy/).

3 Measurement of bed occupancy rates varies across studies. For example, Mennicken et al. [1] compute bed occupancy rates as daily patient count divided by average number of beds in each department, Madsen et al. [14] calculate them as patients assigned to a department over staffed beds in the department at any time and date, Blom et al. [9, 10] consider the hourly proportion of occupied beds, and Friebel et al. [12] use daily inpatients present at midnight over average daily number of beds by quarter. Closer related to our study, Boden et al. [11] compute monthly bed occupancy rates as the ratio of occupied beds over total bed base at midnight. Our aim is to study whether there exists a systematic association between bed occupancy rates and quality and, thus, we calculate bed occupancy rates as average daily number of occupied beds at midnight over the average daily number of available beds by quarter aggregated at annual level (see “Data”).

4 Other studies [29, 30, 49, 50] find that high bed occupancy rates are associated with increases in adverse events occurring in hospitals such as patient falls, pressure ulcers, hospital-acquired pneumonia, hospital-acquired infections, medication errors, complaints, and patient identification errors. Although not focusing on bed occupancy rates, some studies have investigated the relation between efficiency and quality. Several studies use a stochastic frontier approach to estimate hospital efficiency. Deily and McKay [51] show that cost inefficiency is positively associated with mortality for acute hospitals in Florida. McKay and Deily [52] find a lack of association between cost inefficiency and mortality and complication rates for the United States. Martini et al. [53] find that more efficient hospitals in Lombardy (Italy) are associated with higher mortality and lower readmission rates. Laine et al. [54] find no association except for prevalence of pressure ulcers in Finland. Using costs as a proxy for efficiency, some studies find a negative association with quality suggesting a cost-quality trade-off [55, 56] and showing that cost containment and quality improvement might be complements [57, 58]. Others provide evidence that the relationship is U-shaped, with quality reducing costs at low levels of quality and increasing costs at higher levels [58, 59]. Stargardt et al. [60] for Germany and Häkkinen et al. [61] for five European countries (Finland, Hungary, Italy, Norway, and Sweden) address endogeneity of efficiency by using a two-stage residual inclusion model and find mixed results by country and quality measures.

5 In this study, we use the words hospital and trust interchangeably.

6 We have defined *BOR* in terms of beds, occupied or available, *each day*. We could also express the bed occupancy in terms of bed-days over a longer period, e.g. a week, a quarter or a year. If we choose a year, we can define ${B}{O}{R}=\frac{{occupied}\,{bed}-{days}\,{in}\,{a}\,{year}}{{available}\,{bed}-{days}\,{in}\,{a}\,{year}}.$ The volume of patients treated in a year becomes then $\frac{{o}{c}{c}{u}{p}{i}{e}{d}\,{b}{e}{d}-{d}{a}{y}{s}\,{i}{n}\,{a}\,{y}{e}{a}{r}}{{L}{O}{S}}$ giving ${BOR}=\frac{{LOS}\,\times \,{patients}\,{treated}\,{in}\,{a}\,{year}} \, {{a}{v}{a}{i}{l}{a}{b}{l}{e}\,{b}{e}{d}-{d}{a}{y}{s}\,{i}{n}\,{a}\,{y}{e}{a}{r}}=\frac{{L}{OS} \times Y \times 365}{B\,\times \,365}$, which is the same as in Eq. (1).

7 Although beds can be classified as a supply shifter, we include them as a separate variable because they affect directly bed occupancy rates.

8 Bed occupancy is function of these three determinants and therefore we do not include them together due to collinearity.

9 This model involves regressing quality on time-invariant variables and the mean over time and the deviation from their mean of time-variant variables, employing the *xtreg, re* command in Stata.

10 For unbalanced panels, the within coefficients estimated by a hybrid model and by a fixed effects specification should be identical, with slightly different standard errors [38]. We compare these two models in Table 12 in the Appendix.

11 SHMI and condition-specific mortality are risk-adjusted by estimating the expected deaths through a logistic regression controlling for age, sex, admission method, year index, Charlson Comorbidity Index, and diagnosis grouping. Surgical mortality rates are adjusted for age and gender. Emergency readmission rates are risk-adjusted for age, sex, method of admission and diagnosis/procedure. PROMs are risk-adjusted for age, sex, ethnicity, index of multiple deprivation, pre-operative self-assessed health status, comorbidity, patient assistance to complete the questionnaires, living arrangements, disability, primary diagnosis, and years of experiencing symptoms. PROMs for knee replacement exclude living arrangements and years of experiencing symptoms.

12 This is the ratio of observed patients whose death occurred either in-hospital or within 30 days of an operative procedure to expected deaths multiplied by an overall event rate of patients in England. See Appendix 5 in [62] for procedure codes included.

13 It is the ratio of provider’s observed to expected readmissions multiplied by an overall event rate of patients in England.

14 The surveys comprise twelve questions related to patient’s pain and mobility, with five multiple choice answers where 0 denotes greatest severity and 4 least or no symptoms. These answers are then summed up to a single score with 0 indicating the worst possible score and 48 the highest.

15 Bed occupancy cannot be computed by diagnosis or procedure since beds are not labelled by condition (i.e. any patient can occupy any bed).

16 Clinical staff are defined as the sum of doctors, nurses, health visitors, midwives, ambulance staff, and scientific, therapeutic, and technical staff. We compute yearly averages of monthly staff data reported by NHS Digital.

17 Any trust located less than 30 km away from a hospital in question is a competitor since catchment areas overlap. The distance between two hospital trusts is defined as the Euclidean distance between trust headquarters. Hospital competition is calculated using NHS Digital and Open Geography portal datasets.

18 LSOA are small areas (32,844 in England) with an average of 1500 individuals, a minimum of 1000, and a maximum of 3000.

19 Recall that bed occupancy rates (*BOR = LOS* × *Y/B*) can be written as a function of length of stay (*LOS*) and the volume to beds ratio (*Y/B*).

20 Our sample is an unbalanced panel of data mainly due to mergers and acquisitions of hospitals across time and missing data on dependent or control variables.

21 Table 6 in the Appendix presents descriptive statistics of bed occupancy by four thresholds (≤ 85%, 85%–90%, 90%–95%, > 95%) for each quality variable. 15%–25% of the sample lies in the first category (≤ 85%), around 35% in the second and third category, and 7%–12% in the fourth category (> 95%).

22 Table 1 shows that within hospital variation is smaller than between variation for all quality variables in the sample, except for health gains after a hip replacement. This is also the case for bed occupancy rate (Table 1 and Table 8 in the Appendix).

23 Table 3 reports results for Model 5. Estimates for control variables for Model 2 and Model 5 do not dramatically differ. Complete results for Model 2 are available upon request.

24 We only discuss controls associated with hospital quality measures at the 5% significance level.

## Code availability

The authors declare that the software application used in this article is Stata/MP 16.1. The Stata do file with codes used in this article are available upon request.

## Declarations

### Original publication

The authors declare that the manuscript contains original unpublished work and is not being submitted for publication elsewhere at the same time. A contribution in the Spanish Health Economics Association blog was made in March 2021 with a short summary of the study (see http://www.aes.es/blog/2021/03/17/tasas-ocupacion-camas-hospitalarias-calidad-el-caso-de-los-pacientes-agudos/).
